# Extracorporeal Photopheresis: Secreted Factors That Promote Immunomodulation

**DOI:** 10.1097/TXD.0000000000001840

**Published:** 2025-09-02

**Authors:** Jorge H. Garcia-Almeida, Lukas Heger, Holger Hackstein

**Affiliations:** 1 Department of Transfusion Medicine and Haemostaseology, Universitätsklinikum Erlangen, Erlangen, Germany.

## Abstract

**Background.:**

Extracorporeal photopheresis (ECP) is a therapy indicated for various T cell–mediated conditions, including cutaneous T-cell lymphoma (CTCL), graft-versus-host disease (GVHD), and solid organ transplant rejection. ECP comprises the treatment of patients’ leukocytes with 8-methoxypsoralen and ultraviolet-A light followed by autologous reinfusion. ECP exerts therapeutic immune-stimulatory effects in CTCL and immune regulatory effects in GVHD and solid organ transplant rejection. Besides cellular mediators, secreted molecules can contribute to ECP’s therapeutic effect.

**Methods.:**

We conducted a comprehensive review of the literature on ECP-induced secreted factors and their immunomodulatory roles.

**Results.:**

8-Methoxypsoralen/ultraviolet-A treatment drives leukocyte apoptosis, resulting in the release of damage-associated molecular patterns that promote apoptotic cell phagocytosis by dendritic cells (DCs) and promote or impair DC maturation. In CTCL, the increased production of proinflammatory cytokines in photopheresates, including interferon-γ, interleukin (IL)-2, tumor necrosis factor-α, IL-1β, and IL-8, is linked to antitumor responses. Conversely, ECP upregulates anti-inflammatory cytokine production in photopheresates from GVHD patients’ cells. Upon reinfusion of photopheresates containing anti-inflammatory factors, untreated immature DCs are converted to tolerogenic DCs with increased IL-10 and transforming growth factor-β secretion and regulatory T cell–inducing functions. In allograft models, ECP increases IL-4, IL-10, and IL-13, which reduce allograft rejection. Moreover, ECP influences the level of immunomodulatory metabolites and the composition of exosomes. However, further research, for example, using multi-omics approaches, are needed to provide a more comprehensive picture of the ECP-induced secretome and to identify relevant factors that could contribute to ECP’s therapeutic effects.

**Conclusions.:**

ECP induces the release of different pro/anti-inflammatory factors in different preexisting conditions that determine different DC maturation status and immunomodulatory effects.

Extracorporeal photopheresis (ECP) is a leukapheresis-based therapy that obtained approval for the treatment of the cutaneous T-cell lymphoma (CTCL) variant Sézary Syndrome by the Food and Drug Administration in 1988.^1^ In ECP, patients’ leukocytes are separated from plasma and erythrocytes, incubated with the photosensitizing agent 8-methoxypsoralen (8-MOP) and irradiated with ultraviolet-A (UVA) light before reinfusion into the patient. 8-MOP intercalates into the DNA, and upon UVA exposure, leads to the formation of DNA crosslinks and subsequent apoptosis of leukocytes, mainly T cells and natural killer (NK) cells.^2-4^ An ECP cycle is usually defined as a 2-consecutive-day treatment.^5^ Since its introduction, ECP treatment has been extended to other T cell–mediated conditions, including graft-versus-host disease (GVHD) and solid organ transplant rejection, demonstrating high response rates and good safety profiles with no significant unwanted side effects.^1,6-9^ In solid organ transplantation, ECP is beneficial for the prevention and management of heart and lung transplant rejection, and transplanted patients display higher levels of regulatory T cells (Treg ) post-ECP.^10,11^ ECP is thought to increase immunotolerance and survival of heart transplants based on observations in mice.^12^ ECP is also being investigated for its effectiveness in the management of side effects associated with kidney and liver transplantation.^13,14^ In addition, ECP-induced apoptosis in leukocytes could promote tissue reparative macrophages and tissue repair after transplantation.^15,16^

The therapeutic effect of ECP in CTCL was initially attributed to the induction of apoptosis in malignant T cells by 8-MOP and UVA.^17,18^ This mechanism, however, does not explain the therapeutic effect of ECP in other disorders. Evidence suggests that ECP elicits an immunomodulatory mechanism by promoting tolerogenic DCs and Treg in patients with GVHD and chronic heart and lung transplant rejection.^19-23^ Monocytes are more resistant to ECP-induced apoptosis, and those who survive can be activated upon contact with extracorporeal surfaces in the ECP device, leading to their differentiation into immature DCs (iDCs).^24-27^ The dual pro/anti-inflammatory effect of ECP is suggested to rely on interactions of apoptotic cells with 2 kinds of phagocytic mediators: (1) untreated antigen-presenting cells (APCs) that result in immunoregulation and (2) ECP-activated APCs in the presence of proinflammatory mediators, such as IL-1β, that result in immunostimulation.^28^

Although ECP has been used for more than 30 y, its mechanisms of action have not been completely elucidated yet, and the relevance of secreted factors is unknown.^6^ Because many of the disorders in which ECP is effective have an inflammatory etiology and are T cell–mediated, changes in cytokines and other immune cell-secreted factors can play a role in the therapeutic effects of ECP. Moreover, different secreted factors might mediate different therapeutic responses across conditions. It is known that the cytokine environment present during phagocytosis of apoptotic cells and the maturation status of DCs determine the skewing of immune responses.^29-32^ When iDCs process apoptotic cells in a proinflammatory environment, they undergo maturation and are effective at priming effector T cells. Contrarily, in the absence of co-stimulation and inflammation (“danger signals”), apoptotic cell–processing iDCs do not mature and induce antigen-specific immune tolerance. Furthermore, the identification and functional characterization of ECP-induced secreted factors will help us identify biomarkers of ECP’s potency that will open avenues for treatment outcome prediction and personalized treatment plans.^33^

This article revises secreted factor responses of cultured cells, either isolated from patients treated with ECP or incubated with 8-MOP/UVA in vitro, as well as the pro/anti-inflammatory effects of these factors, which are summarized in Figures 1 and 2, respectively.

**FIGURE 1.**
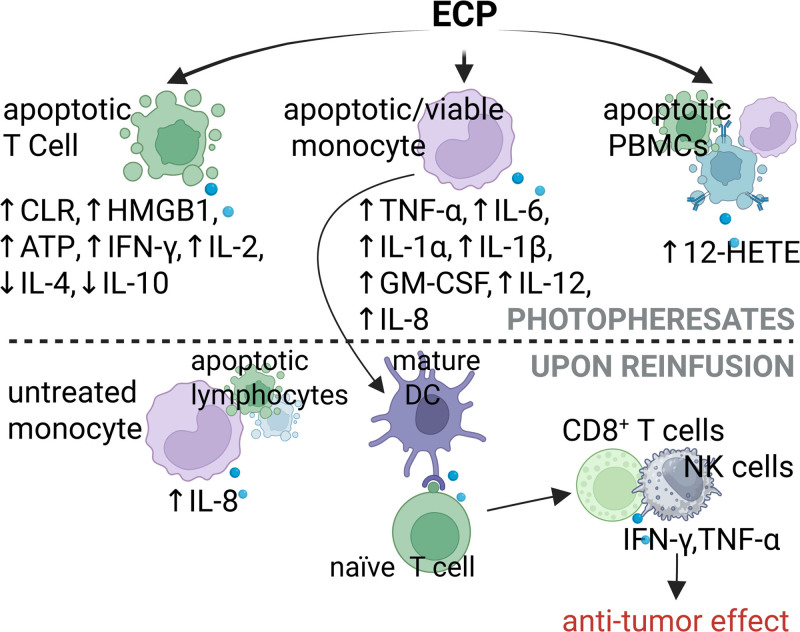
Proinflammatory responses induced by ECP. Surging proinflammatory responses post-ECP have been linked to the antitumor effect beneficial in CTCL. Created in BioRender. Garcia (2025) https://BioRender.com/f59p812. ATP, adenosine triphosphate; CLR, calreticulin; CTCL, cutaneous T-cell lymphoma; DC, dendritic cell; ECP, extracorporeal photopheresis; GM-CSF, granulocyte-macrophage colony-stimulating factor; HETE, hydroxyeicosatetraenoic acid; HMGB1, high mobility group box 1; IFN, interferon; IL, interleukin; NK, natural killer; PBMCs, peripheral blood mononuclear cells; TNF-α, tumor necrosis factor-α.

**FIGURE 2.**
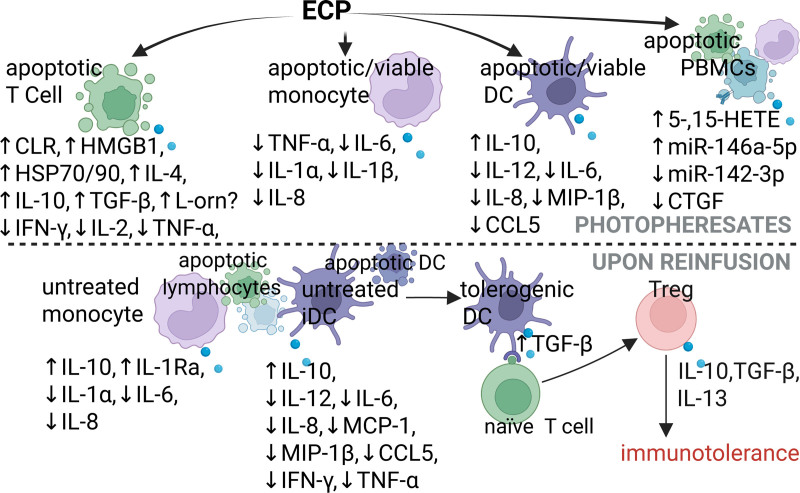
Anti-inflammatory responses induced by ECP. ECP-induced increments in anti-inflammatory responses are associated with immunotolerance in inflammatory conditions. Created in BioRender. Garcia (2025) https://BioRender.com/x50g971. CLR, calreticulin; CTGF, connective tissue growth factor; DC, dendritic cell; ECP, extracorporeal photopheresis; HETE, hydroxyeicosatetraenoic acid; HMGB1, high mobility group box 1; HSP, heat shock protein; IFN, interferon; IL, interleukin; IL-1Ra, interleukin-1 receptor antagonist; L-orn, L-ornithine; MCP-1, monocyte chemoattractant protein-1; MIP-1β, macrophage inflammatory protein-1β; miR, microRNA; PBMCs, peripheral blood mononuclear cells; TGF-β, transforming growth factor-β; TNF-α, tumor necrosis factor-α; Treg, regulatory T cell.

## INDUCTION OF DAMAGE-ASSOCIATED MOLECULAR PATTERNS BY ECP

8-MOP/UVA treatment of melanoma cells in vitro is reported to induce the release of damage-associated molecular patterns (DAMPs) such as calreticulin (CLR), high mobility group box 1 (HMGB1), and extracellular ATP.^34^ Although CLR and ATP can be actively secreted at the early stages of apoptosis, HMGB1 is primarily released passively later in the process, particularly when plasma membrane rupture occurs.^35^ 8-MOP/UVA-induced apoptotic melanoma cells are successfully engulfed by monocytes, resulting in cross-presentation of tumor antigens to CD8^+^ T cells and subsequent initiation of anticancer immunity in this study.^34^ Moreover, anticancer protection was induced by vaccinating syngeneic mice with 8-MOP/UVA-treated melanoma cells and was abolished when melanoma cells were depleted of CLR, HMGB1, or added with an ATP-degrading enzyme. DAMPs mainly function as pro-phagocytic, chemotactic, and/or activatory signals for DCs or their precursors and define an immunogenic variant of apoptotic cell death.^36^ The exposure of the endoplasmic reticulum chaperone CLR on the cell surface is an “eat me” signal that stimulates the phagocytosis of dying cells by DCs.^37,38^ The nonhistone chromatin-binding protein HMGB1 binds to Toll-like receptor 4, activates DCs, and facilitates antigen presentation to T cells.^39^ Extracellular ATP acts as a DC chemoattractant or activation signal by binding to purinergic receptor P2Y2 or purinergic receptor P2X7,^40,41^ activating the NLRP3 inflammasome.^42^ Nevertheless, the contribution of these DAMPs to the therapeutic effect of ECP was not tested in this study. Notably, the antitumor protection in mice was observed with higher UVA doses (4 J/cm^2^) than those used in the clinic (1–2 J/cm^2^).^43^

A study using mixed leukocyte reaction (MLR)–activated alloreactive T cells as an in vitro model of GVHD revealed that 8-MOP/UVA treatment of alloreactive T cells from healthy donors induced a partially immunogenic phenotype, as dying T cells increased CLR expression and the release of HMGB1 and the heat shock proteins (HSPs) 70 and 90, but not ATP.^44^ This phenotype allowed phagocytosis by DCs and monocytes but is not followed by DC maturation and allogeneic naive T-cell proliferation. CLR expression was also increased in 8-MOP/UVA-treated GVHD patients’ T cells. Interestingly, HSP70 release can also exert anti-inflammatory effects by turning DCs toward tolerogenic DCs with increased IL-10 production and reduced tumor necrosis factor-α (TNF-α), CD86, and major histocompatibility complex class II expression, leading to Treg induction.^45^ Moreover, treatment with HSP70 can inhibit acute skin allograft rejection.^46^

## CYTOKINE RESPONSES INDUCED BY ECP

Cytokines are secreted actively and differentially in different diseases after ECP. Increments in proinflammatory cytokines in serum post-ECP are associated with clinical responses in CTCL patients, whereas increased anti-inflammatory cytokines are suggested to be beneficial in inflammatory conditions such as GVHD and systemic sclerosis.^47-50^ Some studies have reported direct cytokine responses in supernatants of cells cultured post-ECP that mimic cytokine responses within photopheresates, whereas others have focused on indirect cytokine responses in supernatants of untreated cells co-cultured with ECP-treated cells that model cytokine responses upon reinfusion of photopheresates. No studies assessing cytokine responses in serum were included in this article.

### Proinflammatory Cytokine Responses in Photopheresates

Apoptotic T cells from both CTCL patients and healthy donors induced, respectively, by ECP (after 1 y of treatment) or 8-MOP/UVA in vitro are reported to increase interferon (IFN)-γ transcription and production and interleukin (IL)-2 production while decreasing IL-4 transcription and production as well as IL-10 production.^47,51,52^ It has been shown that the T helper type 1 (Th1) proinflammatory cytokines IFN-γ and IL-2 activate the tumoricidal effects of CD8^+^ T cells.^53^ IFN-γ has been reported to benefit patients with CTCL.^54^ Apoptotic melanoma cells induced by 8-MOP/UVA depicted upregulation of IFN-β production that displays proinflammatory effects during inflammation by inducing monocyte chemoattractant protein-1 (MCP-1) and interferon gamma-induced protein 10 and anti-inflammatory effects in the absence of a proinflammatory environment via IL-10 induction.^34,55^

Monocytes from CTCL and systemic sclerosis patients treated with ECP (after an average of 32 mo of monthly treatment) upregulate levels of TNF-α and IL-6 but not those of IL-1α or IL-1β.^52,56^ However, TNF-α was undetectable in the serum of patients until 18 h post-ECP.^56^ Meanwhile, lipopolysaccharide (LPS)-, CD2-, CD3-, and CD28-stimulated peripheral blood mononuclear cells (PBMCs) from ECP-responding CTCL patients (after 27 weekly cycles) increased levels of TNF-α, IL-1α, IL-1β, and granulocyte-macrophage colony-stimulating factor.^56,57^ The antitumor effects of TNF-α involve enhancement of monocyte cytotoxicity and NK cell activities,^58^ and may be important in the differentiation of cytotoxic T lymphocytes.^59^ TNF-α may also exert direct tumoricidal activity against T-cell lymphoma lines.^60^ Interestingly, ECP-treated monocytes from both CTCL (after 1 y of treatment)^47^ and GVHD patients (after 6 mo of 11 ECP cycles)^61^ upregulate IL-12, which can exert both proinflammatory and anti-inflammatory functions. On the one hand, IL-12 promotes Th1 responses by IFN-γ production in T helper lymphocytes, NK cell cytotoxicity, and CD8^+^ T-cell responses.^62^ On the other hand, IL-12 can enhance the expression of the immunoregulatory cytokine IL-10 in Th1 cells as negative feedback for the immune responses induced by IL-12.^63,64^ IL-12 therapy is also reported to induce lesion regression in CTCL.^62^ Healthy donor’s monocytes treated with 8-MOP/UVA upregulated IL-8 and IL-1β.^52,65^ IL-1β is reported to be secreted by DC to induce, together with IL-2, tumor-specific cytotoxic CD8^+^ T cells,^40,66^ as well as to inhibit Treg.^67^

### Proinflammatory Cytokine Responses Upon Reinfusion

Co-cultures of untreated cells and autologous ECP-induced apoptotic T cells have been performed to mimic responses upon reinfusion of ECP-treated cells. Untreated monocytes from healthy donors exposed to autologous 8-MOP/UVA-induced apoptotic CD4^+^ T lymphocytes secreted higher quantities of IL-8 in the presence of anti-CD3/CD28.^68^ IL-8 has been described to promote the recruitment of cytotoxic T cells around tumor antigen-bearing DCs into which monocytes differentiated, resulting in induction and activation of tumoricidal T cells.^52^

### Anti-inflammatory Cytokine Responses in Photopheresates

In T cells from GVHD patients treated with ECP (after 12 monthly cycles), IL-4 and IL-10 production increases, whereas IFN-γ, IL-2, and TNF-α production decreases.^48,69-71^ IL-4 and IL-10 produce a negative feedback on Th1 differentiation and suppress Th1 and cytotoxic T-cell responses, favoring engraftment.^48,72^ Decrements in IFN-γ, IL-2, and TNF-α secretion were also found in T cells isolated from CTCL patients after ECP (after 12 monthly cycles).^69,70^ Healthy donor’s PBMCs treated with 8-MOP/UVA upregulate the transcription of transforming growth factor-β (TGF-β) and IL-10, and TGF-β was suggested to be partly responsible for the inhibition of allogeneic T-cell proliferation in MLR assays.^73^

8-MOP/UVA- and ECP-treated monocytes (after 12 monthly cycles) have shown decreased levels of the proinflammatory cytokines TNF-α, IL-6, IL-1α, IL-1β, and IL-8 in GVHD^69,71^ and TNF-α, IL-6, IL-1β, and IL-8 in psoriasis and healthy donors.^74^ A study with CTCL patients’ monocytes also reported decreased TNF-α production post-ECP (after 12 monthly cycles).^69^ These monocyte responses might be, at least in part, the result of the aforementioned increased IL-10 and TGF-β production by ECP-treated T cells within photopheresates. IL-10 is indicated to inhibit the transcription of TNF-α, IL-1β, IL-6, IL-1α, IL-8, granulocyte-macrophage colony-stimulating factor, and granulocyte-colony stimulating factor in LPS-stimulated monocytes.^75,76^ Accordingly, TGF-β can increase its own transcription and inhibit TNF-α translation in macrophages^77,78^ and IL-1 release by bone marrow progenitor cells.^79^

DCs from GVHD patients treated with ECP (after 3 monthly cycles) have shown an increase in IL-10 production.^26^ 8-MOP/UVA-treated DCs from healthy donors also elevated IL-10 while reducing levels of IL-12, IL-6, IL-8, and the chemokines MCP-1, macrophage inflammatory protein-1β, and CCL5, but not TNF-α nor IFN-γ.^80^

### Anti-inflammatory Cytokine Responses Upon Reinfusion

In co-cultures of untreated monocytes from GVHD patients and autologous lymphocytes treated with ECP (after 6 mo of 11 cycles, after 12 monthly cycles, or until resolution of GVHD in 4-w k cycles) as in vitro models of reinfusion, IL-10 and IL-1 receptor antagonist (IL-1Ra) transcription and production are upregulated, whereas IL-1α, IL-6, and IL-8 production is downregulated.^61,71,81^ TNF-α remained unchanged, whereas IL-12 and IL-1β levels were either reduced or remained unchanged amongst studies. Notably, IL-10 mediates IL-1Ra transcription in monocytes in this co-culture model.^81^ Similarly, TGF-β can increase the production of IL-1Ra as evidenced in bone marrow progenitor cells.^79^ Therefore, the increased IL-1Ra production by monocytes upon reinfusion could be mediated by the increased IL-10 and TGF-β, secreted by apoptotic T cells within photopheresates. Interestingly, recombinant IL-1Ra is suggested to be more effective than recombinant IL-10 in protecting against murine GVHD.^82,83^

DC responses upon reinfusion have been studied in various in vitro models. Untreated DCs from GVHD patients or healthy donors co-cultured with autologous lymphocytes treated with ECP (after at least 3 mo of weekly cycles) or 8-MOP/UVA, respectively, display surging production of IL-10.^80,84^ Healthy donor’s DC response was accompanied by reduced production of IL-12, IL-6, IL-8, MCP-1, macrophage inflammatory protein-1β, CCL5, IFN-γ, and TNF-α.^80^ Autocrine IL-10 prevents spontaneous DC maturation and converts immature DCs into tolerogenic DCs with increased IL-10 production.^85^ Moreover, IL-10-treated DCs have been reported to induce alloantigen- or peptide-specific Treg that suppress the proliferation of syngeneic T cells in a dose-dependent and cell-to-cell contact-dependent manner in vitro.^86,87^ In another co-culture model, UVA-induced apoptotic DCs convert autologous untreated immature DCs into tolerogenic DCs with increased TGF-β1 secretion in both LPS-stimulated and unstimulated conditions.^88^ Importantly, the immune regulatory effects of ECP are thought to be mediated mainly through Treg and the cytokines IL-10 and TGF-β.^85,89^ IL-10 is indicated to mediate antigen-specific Treg induction in a murine model of contact hypersensitivity following infusion of syngeneic 8-MOP/UVA-treated leukocytes.^90^ TGF-β can mediate the induction of Treg in vitro^88^ and plays a critical role in Treg differentiation.^91^ Treg consequently secrete IL-10 and TGF-β upon antigen-specific stimulation in the presence of APCs.^90,92^ In addition to Treg, IL-10-secreting regulatory B cells (Bregs) are upregulated in mice after infusion of allogeneic 8-MOP/UVA-treated lymphocytes.^93^ Gray et al^94^ reported that Bregs induced by infusion of syngeneic apoptotic thymocytes in mice increase IL-10 secretion, suppress effector T cells, and induce IL-10-secreting Treg.

### Anti-inflammatory Cytokine Responses in Allograft Models

The immunoregulatory capacity of ECP has been evidenced by a reduction in allogeneic effector T cell numbers and increased anti-inflammatory cytokine responses in MLR assays as in vitro allograft models.^46,73,95^ It is reported that the co-culture of 8-MOP/UVA-induced apoptotic PBMCs and untreated allogeneic PBMCs displays a reduction in IFN-γ, TNF-α, and IL-6.^73^ When only 8-MOP/UVA-treated immature monocyte-derived DCs from healthy donors are co-cultured with allogeneic naive T cells, upregulation of IL-10, IL-4, and IL-13 levels besides IFN-γ and TNF-α reduction is observed, which is partially reversed by the addition of IL-12.^95^ IL-10, IL-13, and IL-4 levels are reported to prolong allograft survival^96-98^ that is also correlated with reduced IFN-γ levels.^99^ IL-13 is also suggested to induce and maintain allograft tolerance by inhibiting dendritic cell and macrophage activation via IL-12 and TNF-α mRNA reduction.^97^ Moreover, IL-4, IL-13, IL-10, and IL-33 are reported to promote M2 reparative macrophages.^15^ Lack of IL-12 secretion by DCs, as described previously for both DCs in photopheresates and untreated DCs upon reinfusion, is needed for the promotion of Th2 cells and Th2 cytokine production.^100^ Remarkably, ECP induces anti-inflammatory cytokine responses when only the donor alloantigen is treated with 8-MOP/UVA. In a rat model of cardiac transplantation, recipient DCs loaded with donor 8-MOP/UVA-treated splenocytes elevated IL-10 and IL-4 while reducing IFN-γ in co-cultures with recipient T cells, induced recipient’s Treg, and reduced cardiac allograft rejection.^101^ Therefore, loading recipient DCs with ECP-treated donor antigens seems key to counteract anti-donor alloreactivity.

## OTHER SECRETED FACTORS INDUCED BY ECP

ECP-induced apoptosis influences transcriptional, posttranscriptional, and translational systems correspondingly, resulting in relevant changes in protein and metabolic levels.^102^ Therefore, metabolite and protein analyses in ECP-treated cells are important. However, such studies are limited, and thus far, no metabolome or proteome analyses have been reported. Merlin et al^103^ found that in vitro 8-MOP/UVA treatment of PBMCs from GVHD patients and healthy donors, stimulated with the lymphocyte-stimulant phytohemagglutinin, induced the synthesis of L-ornithine (L-orn) from arginine in the polyamine pathway, which in turn upregulates IL-10 and downregulates TNF-α and IFN-γ. L-ornithine is passively released from degrading apoptotic cells and can inhibit alloreactive proliferation in the MLR setting.^104^ Another study revealed that ECP (24 h after 1 cycle) or 8-MOP/UVA treatment increases the levels of hydroxyeicosatetraenoic acids (HETEs), particularly 5-HETE, 8-12-HETE, and 15-HETE, in plasma and PBMCs of patients with CTCL and progressive systemic sclerosis and healthy donors.^105^ HETEs are actively secreted metabolites of arachidonic acid and markers of stable lipid peroxidation. 5-HETE and 15(S)-HETE are reported to be anti-inflammatory. 5-HETE induces antioxidative enzymes in resting vascular endothelial cells,^106^ which inhibit expression of adhesion molecules that mediate leukocyte recruitment,^107^ whereas 15(S)-HETE inhibits platelet reactivity^108,109^ and leukotriene B4-induced chemotaxis of polymorphonuclear neutrophils in vitro.^110^ On the other side, the proinflammatory 12(S)-HETE induces platelet activation^111^ and monocyte binding to activated endothelial cells, which results in leukocyte migration.^112^

Exosomes might also harbor relevant secreted factors, because exosomes are actively secreted by apoptotic cells and are involved in intercellular communication by carrying nucleic acids, proteins, lipids, and metabolites that modulate immune cells, potentially influencing ECP’s effectiveness.^113^ Bozzini et al^114^ recently published the first study about exosomes post-ECP. Here, exosomes from chronic lung allograft dysfunction patients’ mononuclear cells treated with ECP (after 6 mo in 10 cycles) and stimulated with LPS displayed microRNA-142-3p (miR-142-3p) downregulation, miR-146a-5p upregulation, and a decrease in connective tissue growth factor, which plays a key role in posttransplant fibrogenesis. miR-142-3p downregulation is reported to confer Treg suppressive functions^115^ and might also reduce IL-6- and TNF-α-mediated proinflammatory activity in monocyte-derived DCs and increase Treg numbers.^116^ miR-146a expression is increased in activated Treg ^117^ and LPS-stimulated monocytes.^118^ miR-146a plays a role in the negative regulation of myeloid cells through restriction of TNF receptor-associated factor 6 stimulation and interleukin receptor-associated kinase 1/2-mediated signaling in inflammation.^119,120^ Thus, ECP induces immunomodulatory metabolites as well as changes in the content of exosomes toward immunoregulation.

## DISCUSSION

The mechanisms of action of ECP rely not only on interactions between apoptotic cells with APCs upon reinfusion of photopheresates but also on factors secreted before and after those interactions. Thus far, several cytokines, DAMPs, metabolites, miRs, and growth factors have been associated with the skewed immune responses observed post-ECP.

The DAMPs CLR, HMGB1, and ATP are released by melanoma cells after 8-MOP/UVA treatment, which jointly promote phagocytosis by DC, DC activation, and antimelanoma protection in mice.^34^ CLR, HMGB1, and HSP70/90, but not ATP, are induced by ECP in an in vitro model of GVHD and together mediate phagocytosis by DCs but impair DC maturation.^44^ These studies suggest that ECP induces common DAMPs (CLR, HMGB1) across conditions that allow phagocytosis of apoptotic cells by APCs, but also specific DAMPs under different conditions that influence DC maturation differently and might determine the differential therapeutic effect of ECP. Nevertheless, the individual contribution of these and other DAMPs to ECP’s therapeutic effect is yet to be studied. It is important to note that evidence supporting the induction of immunogenic cell death by ECP, besides apoptosis, is limited. Different cell death mechanisms might target different cell types, releasing different secreted mediators that mediate the phagocytosis of dying cells.

CTCL patients’ T cells and monocytes showed that ECP reduces anti-inflammatory cytokines while increasing the proinflammatory cytokines IFN-γ, IL-2, TNF-α, IL-1β, and IL-8^47,51,52,56,57^ that are reported to induce antitumor responses.^53,56,58-60^ Conversely, ECP downregulates proinflammatory cytokines and upregulates several anti-inflammatory cytokines in T cells, monocytes, and DCs from GVHD patients and allograft models, including IL-10^26,48,69-71^ and TGF-β^73,88^ with Treg-promoting functions.^88,90,91^ This is in line with the fact that apoptotic cells release TGF-β, as well as that monocytes and macrophages engulfing apoptotic cells release TGF-β and IL-10.^121-123^ Thus far, cytokine analysis post-ECP is limited to targeted cytokines, excluding other apoptosis-induced cytokines that could be mediating ECP’s effects. For instance, PBMCs rendered apoptotic by TNF-α and Staurosporine treatment can secrete IL-38, which reduces IL-6 and IL-8 production in macrophages.^124^ Furthermore, healthy donors’ cells treated with ECP display either a proinflammatory or anti-inflammatory response.^51,52,65,68,73,74,80^ It is unclear how the preexisting inflammatory status determines different beneficial responses post-ECP. It is suggested that treated iDCs from photopheresates mature and activate effector T cells in the presence of proinflammatory mediators such as IL-1β, whereas untreated iDCs do not mature and induce immune tolerance in the absence of inflammation and DAMPs.^28,31,32^ This could explain the increased production of anti-inflammatory cytokines upon reinfusion in healthy donor’s DCs, in which preexisting inflammation is absent. Importantly, the pro/anti-inflammatory factors secreted within photopheresates might provide or induce the inflammatory environment required for the promotion/prevention of DC maturation. For instance, IL-10, which is produced by T cells and DC within photopheresates, is reported to prevent DC maturation and convert immature DCs into tolerogenic DCs that induce Treg .^85-87^ However, it remains to be investigated how ECP induces different pro/anti-inflammatory factors in photopheresates under different pre existing conditions that affect DC maturation differentially. Moreover, no studies have assessed the effects of the ECP-triggered cytokine milieu on specific immune cells after reinfusion of ECP-treated cells, which could advance our knowledge of ECP’s mechanisms of action.

L-ornithine is an anti-inflammatory metabolite passively released by ECP from GVHD patients’ and healthy donors’ apoptotic PBMCs^103^ that can inhibit alloreactivity.^104^ However, it is still unknown what and how cell subsets secrete L-ornithine upon ECP. HETEs are actively secreted post-ECP in plasma and by PBMCs of patients with CTCL, systemic sclerosis, and healthy donors.^105^ Due to HETEs’ dual proinflammatory^111,112^ and anti-inflammatory roles,^106,108-110^ further research is needed to dissect whether and what HETEs are differentially induced by ECP in CTCL and inflammatory conditions. Although not in the context of ECP, Medina et al^125^ identified >100 metabolites released from apoptotic Jurkat T cells, thymocytes, and macrophages after Fas, ultraviolet C, or toxin-mediated apoptosis, and 6 metabolites were shared amongst cell types, including adenosine monophosphate, guanosine monophosphate, creatine, glycerol 3-phosphate, ATP, and spermidine. Spermidine is produced downstream of ornithine in the polyamine pathway, which remained unchanged in this study. Significantly, a mixture of spermidine, guanosine monophosphate, and inosine monophosphate reduced inflammation in a lung transplant rejection mouse model.^125^ Apoptotic cell-released metabolites are also hypothesized to contribute to a regenerative tissue environment.^16,125,126^ After uptake of apoptotic human Jurkat T cells in vitro, LR73 hamster phagocytic fibroblasts release lactate, a natural byproduct of aerobic glycolysis that upregulates anti-inflammatory macrophage genes such as *Tgfb*, *Il10*, and anti-inflammatory/M2-like markers.^127^ Postefferocytosis macrophages can additionally trigger phosphatidylserine-dependent cholesterol efflux as a homeostatic response to the apoptotic cell-derived increase in cholesterol.^128^ Moreover, ECP modulates the expression of exosomal miRs in chronic lung allograft dysfunction patients’ mononuclear cells^114^ with documented anti-inflammatory roles.^115,116,119,120^ Exosome analyses post-ECP in GVHD and CTCL are lacking. It is known, however, that apoptotic PBMCs induced by gamma irradiation express higher quantities of exosomes, proangiogenic proteins, and oxidized phospholipids, which jointly attenuated ischemic damage in a pig model of acute myocardial infarction.^129^ Furthermore, responses of neutrophils and platelets, which are also present in buffy coats exposed to ECP and can secrete high quantities of molecules, need to be further explored. A recent study revealed that 8-MOP/UVA treatment in vitro does not alter levels of the platelet soluble activation markers P-selectin or platelet factor 4^130^ but did not assess other platelet-secreted molecules. Thus, multi-omics approaches, including proteome-, metabolome-, lipidome-, and transcriptome-wide approaches, are needed to obtain a complete picture of ECP’s secretome.

Taken together, ECP acts primarily through the physical interaction of apoptotic cells with ECP-activated or untreated DCs, leading to the release of immunomodulatory factors. Cellular and secreted mediators could have synergistic immunomodulatory and tissue regenerative effects that might be determined by the inflammatory environment in which apoptotic cells are phagocytosed. How ECP induces different pro/anti-inflammatory factors in photopheresates under different preexisting conditions remains to be elucidated. Because ECP affects multiple cells in circulation, understanding what and how secreted factors contribute differentially to the desired response will help us identify biomarkers of potency and novel therapeutic targets, which will allow for optimization of ECP treatment and new therapeutic strategies.^33^
